# Variation in expression of HMW1 and HMW2 adhesins in invasive nontypeable *Haemophilus influenzae *isolates

**DOI:** 10.1186/1471-2180-8-83

**Published:** 2008-05-29

**Authors:** Maria Giufrè, Alessandra Carattoli, Rita Cardines, Paola Mastrantonio, Marina Cerquetti

**Affiliations:** 1 Department of Infectious, Parasitic and Immune-mediated Diseases, Istituto Superiore di Sanità, Viale Regina Elena 299, 00161 Rome, Italy

## Abstract

**Background:**

Among surface antigens of nontypeable *Haemophilus influenzae *(NTHi), the HMW1 and HMW2 proteins are the major adhesins promoting colonization of the upper respiratory tract. Since they are potential vaccine candidates, knowledge concerning variation in HMW proteins expression among clinical isolates is of great interest. In this study, expression of *hmw1A *and *hmw2A *genes was evaluated by quantitative real-time reverse transcription-PCR in 3 NTHi invasive isolates (strains 56, 72, 91) and in the prototype strain 12. Number of 7-bp repeats within the *hmwA *promoters and presence of HMW proteins by Western blotting were also determined.

**Results:**

Results showed that gene transcription varied not only among different isolates but also between the *hmw1A *and *hmw2A *genes from the same isolate. Compared to that found in prototype strain 12, up-regulation of the *hmw1A *gene expression was found in strain 56, down-regulation of both *hmw1A *and *hmw2A *genes transcripts was observed in strain 72 whereas the two *hmwA *genes appeared differentially expressed in strain 91 with the *hmw1A *transcript enhanced but the *hmw2A *transcript reduced.

**Conclusion:**

Increasing numbers of 7-bp repeats within the *hmwA *promoters generally correlated with decreased amounts of mRNA transcript, however additional control mechanisms contributing to modulation of *hmw1A *gene seem to be present.

## Background

Nontypeable *Haemophilus influenzae *(NTHi) is responsible for respiratory tract infection and, especially in adults with underlying conditions, invasive disease such as septicemia and meningitis [1,2]. Several adherence factors, including HMW1/HMW2 proteins, Hia, Hap and hemaglutinating pili, promote colonization of the upper respiratory tract, a prerequisite for disease [3]. Whether specific adhesins play or not a role during the dispersion of the microorganism within the respiratory tract or to sterile sites is currently under debate. Over the past decade, it has become increasingly evident that bacteria have evolved a number of mechanisms to survive and proliferate in hosts during the different stages of infection by regulating gene expression [4]. In almost 80% of NTHi clinical isolates, HMW1/HMW2 proteins are the major adhesins [5]. The HMW1 and HMW2 proteins are encoded by separate chromosomal loci, *hmw1 *and *hmw2*, respectively, each containing an *hmwA *gene, which encodes the structural protein, and two accessory genes, called *hmwB *and *hmwC*, encoding proteins involved in processing and surface localization of the HMW adhesins [6,7]. The sequences of the *hmw1A *and *hmw2A *genes are identical for the first 1,259 bp and thereafter partially diverge [6]. The HMW1 and HMW2 proteins exhibit different cellular binding specificities [5]. The two different binding domains are localized near the N terminus of the mature proteins in a region of maximal sequence dissimilarity between each other [8]. In particular, the 124 amino acids between residues 114 and 237 in mature HMW1 and the 125 amino acids between residues 112 and 236 in mature HMW2 have been found to be essential for full-level adhesive activity [8]. These essential regions have been previously referred to as the HMW1 and HMW2 core-binding domains and the encoding sequences as the *hmw1A *and *hmw2A *core-binding domain sequences [9].

Although the HMW adhesins mediate the attachment of the bacterium to human epithelial cells, they also have an important role in human host immunity being a target of naturally acquired human opsonophagocytic antibodies [10]. Given the importance of HMW adhesins in both NTHi pathogenicity and host immune response, they have gained significant potential as NTHi vaccine candidates.

Analyzing laboratory variants of the wild-type strain 12, Dawid and colleagues demonstrated that the expression of HMW1 and HMW2 adhesins undergo phase variation with an inverse step-wise relationship between the number of 7 bp tandem repeats, located in the *hmw1A *and *hmw2A *promoters, and the level of protein expression [11]. Northern blot analysis of transcripts from the strain 12 variants carrying different repeat numbers in the *hmw2A *promoter demonstrated that the amount of the *hmw2A *gene transcript was maximal in the variant with 15 repeats and decreased progressively in variants with increasing number of repeats [10]. No further investigations have been reported in the literature on variation in HMW adhesins expression among NTHi clinical isolates. In our previous report, we found some invasive NTHi isolates containing both *hmw1A *and *hmw2A *genes but expressing only one or even no reactive HMW protein [9]. In this study, the expression of the *hmw1A *and *hmw2A *genes was evaluated by quantitative real-time reverse transcription-PCR (qRT-PCR) in 3 invasive NTHi isolates and in the prototype strain 12. Moreover, in each isolate, the presence of detectable HMW proteins as well as the number of the repeats within the *hmw1A *and *hmw2A *promoters were investigated.

## Results

### Analysis of repeat number within the *hmw1A *and *hmw2A *promoters

To determine the exact number of the 7-bp tandem repeats in the *hmw1A *and *hmw2A *promoters, amplicons including the *hmw1A *and *hmw2A *gene upstream regions were sequenced in each NTHi isolate (strains 56, 72, 91 and 12). As shown in Table 1, a wide variation in repeat number, ranging from 9 to 28 repeats, was observed. Repeat number strongly varied not only among different isolates but also between the *hmw1A *and *hmw2A *genes from the same isolate (see strains 56 and 91). Since variation in repeat number has been reported to occur during growth in vitro [11], several individual colonies for each isolate were analyzed. Moreover, each isolate was tested for in vitro stability of the number of repeats within *hmwA *promoters by sub culturing it up to ten passages and determining the repeat number at both the fifth and tenth passages. A very slight variation in number (± 1) was found among original colonies from the same isolate as well as among derivatives from the original colonies (data not shown). Nevertheless the results reported in this study refer to the number of repeats from the same colony used as inoculum for RNA purification and qRT-PCR tests.

**Table 1:** Number of 7 bp tandem repeats within the *hmw1A *and *hmw2A *promoters in comparison with HMW protein expression as determined by Western blot analysis.

Strain	Number of 7 bp repeats *hmw*1A promoter	Number of 7 bp repeats *hmw*2A promoter	HMW reactivity (Number of protein bands)		
			
			Polyclonal 28D	3D6 MAb	AD6 MAb
12	16	16	2 bands	2 bands	1 band
56	15	9	2 bands	2 bands	2 bands
72	28	28	-	-	-
91	13	20	1 band	1 band	1 band

- no protein band detected

### Gene expression analysis by qRT-PCR

**Figure 1:**
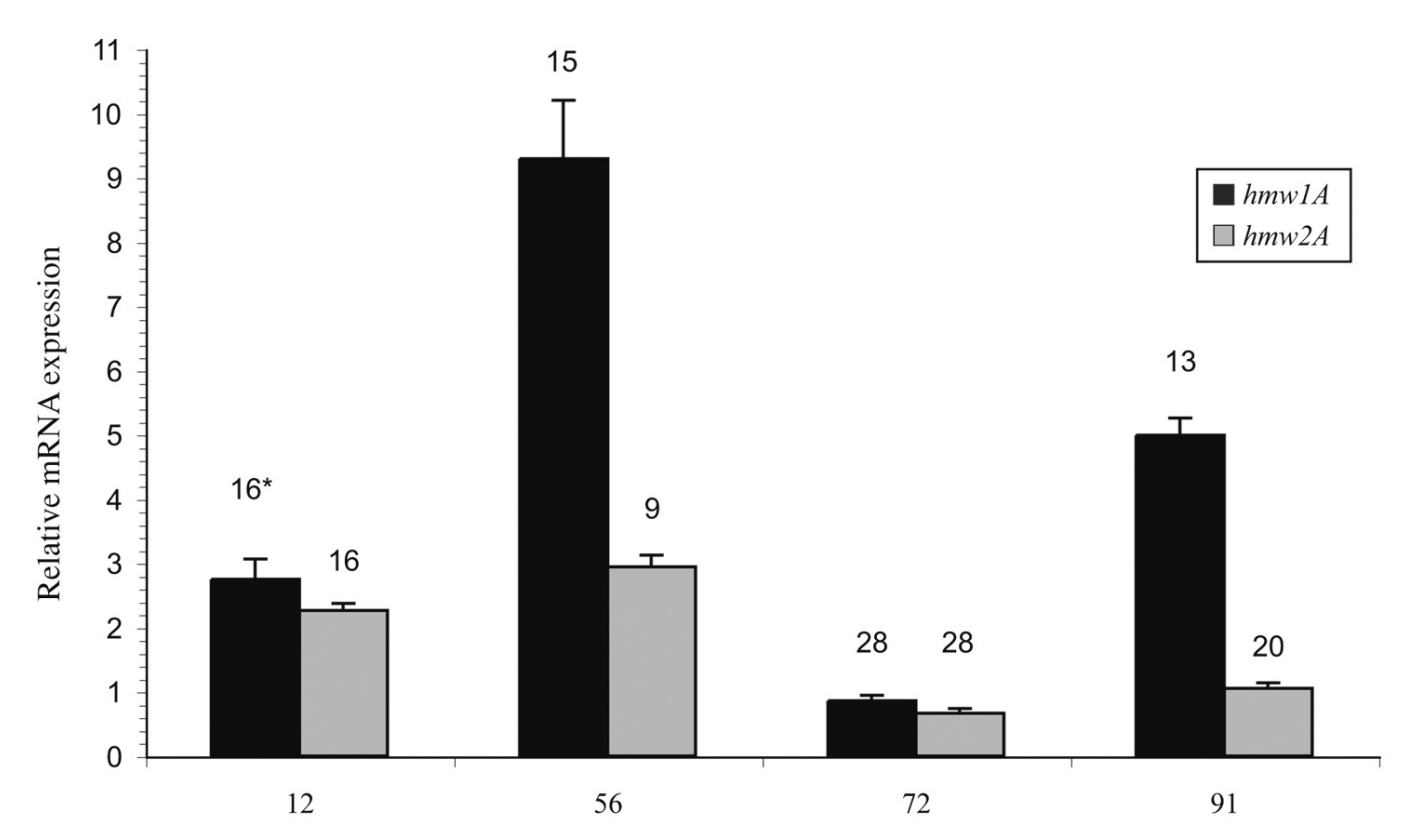
**mRNA expression of the *hmw*1A and *hmw*2A genes from the NTHi strains 12, 56, 72 and 91 determined by qRT-PCR**. The amount of target *hmw*1A and *hmw*2A mRNA transcripts was normalized to the amount of *gyrA *mRNA. Data are presented as means + SEM (error bars) of three experiments performed in triplicate. * Number of repeats within the *hmwA *promoters is indicated at the top of each bar in the graph.

The expression of the *hmw1A *and *hmw2A *genes was determined by qRT-PCR. Data from qRT-PCR experiments were analyzed by relative quantification: the amount of target *hmw*1A and *hmw*2A mRNA transcripts was normalized to the amount of standard *gyrA *mRNA transcript. The relative mRNA expression of the *hmw1A *and *hmw2A *genes from strains 56, 72, 91 and prototype strain 12 is shown in Fig. 1. Compared to the *hmw1A *mRNA transcript from the reference strain 12, both *hmw1A *and *hmw2A *transcripts were particularly reduced (approximately three and fourfold, respectively) in strain 72, which exhibited the highest number of repeats (28 repeats) in both *hmw1A *and *hmw2A *promoters. Differential expression of the *hmw1A *and *hmw2A *genes was found in strain 91, in which the *hmw1A *transcript was slightly increased whereas the *hmw2A *transcript was almost threefold less. Finally, the expression of the *hmw1A *transcript appeared up-regulated (an over threefold increase) in strain 56, although the corresponding promoter contained 15 repeats, which is a value close to that found in the *hmw1A *promoter from the reference strain 12.

### HMW protein expression

**Figure 2:**
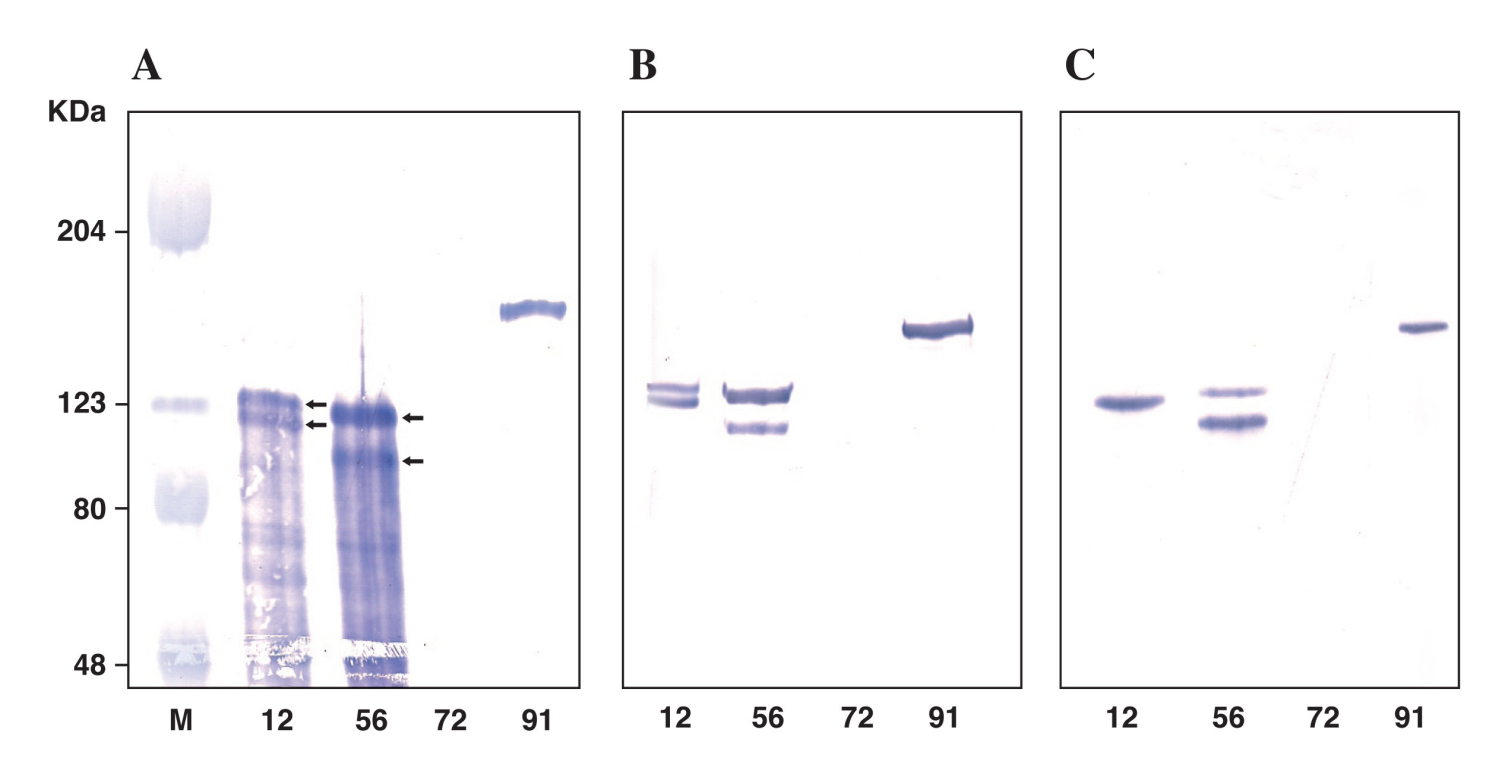
**Western blot analysis of the whole-cell proteins extracted from NTHi strains 12, 56, 72 and 91 probed with the 28D rabbit polyclonal antiserum (panel A), the 3D6 MAb (panel B) and the AD6 MAb (panel C)**. Whole-cell proteins were separated by SDS-PAGE and Western blot analysis was performed as described in Methods. Strain code numbers are indicated below lanes. M, Prestained SDS-PAGE standards (High Range). Arrows in panel A indicate the two HMW proteins of strains 12 and 56 recognized by the 28D polyclonal antiserum, that reacted with a single band in strain 91 and no band in strain 72.

The presence of HMW-reactive proteins was assessed by Western blotting using the 28D rabbit polyclonal antiserum and the 3D6 and AD6 MAbs (Figure 2; Table 1). Since our antisera (both MAbs and polyclonal antiserum) cannot distinguish between HMW1 and HMW2 adhesins (with the partial exception of the AD6 MAb recognizing better the HMW2 than the HMW1), we could only assess the presence/absence of one or two HMW-reactive protein bands detected by each antiserum.

As expected, the prototype strain 12 exhibited two HMW protein bands with both 28D antiserum and 3D6 MAb but a single band with the AD6 MAb, which, in this isolate, reacts preferentially with HMW2. Among our invasive NTHi isolates, strain 56 showed two reactive bands with all antisera used, confirming the expression of both HMW1 and HMW2 proteins. Strain 91 displayed only one reactive band (reasonably the HMW1 protein according to the mRNA analysis), but the presence of two co-migrating HMW proteins of similar size could not be ruled out. No HMW-reactive band was detected in strain 72, irrespective of the antiserum used, suggesting a low level of expression of both HMW proteins.

## Discussion

During natural disease in human, *H. influenzae *is exposed to a variety of environmental conditions, ranging from the nasopharynx to the middle ear or, if invasive disease occurs, to the bloodstream. Each *hmw*-positive NTHi isolate actually possesses two different *hmwA *genes as a result of a gene duplication event that occurred early in its evolution [12]. With this picture, it is reasonable to suppose that expression of *hmwA *genes is modulated during the different stages of disease. Although a previous study on variants from the wild-type strain 12 demonstrated that expression of the HMW1 and HMW2 adhesins varies according to the number of 7-bp tandem repeats in the *hmw1A *and *hmw2A *promoters [11], no further data evaluating changes in HMW expression among clinical NTHi isolates are reported in the literature. In this study, three invasive NTHi isolates previously found to possess both *hmw1A *and *hmw2A *genes [9] were chosen and analyzed in comparison with the prototype strain 12. To evaluate quantitative expression of *hmw1A *and *hmw2A *genes we used qRT-PCR, a highly sensitive method able to accurately measure mRNA transcript level. Results showed that *hmwA *genes transcription varied not only among different isolates but also between the *hmw1A *and *hmw2A *genes from the same isolate, although variations did not exceed a three or fourfold decrease or increase compared with the *hmw1A *gene expression measured in the reference strain 12. The *hmw1A *gene expression was generally higher than that of the *hmw2A*. Analyzing the repeat number within the promoters, the mRNA level and the HMW-reactive proteins expressed, we observed that overall expression of the *hmwA *genes is greatly influenced by the number of repeats within the promoter. The expression was particularly low when the number of repeats was 20 or 28 (*hmw1A *promoter of strain 91 and both *hmwA *promoters of strain 72), confirming results previously reported [11]. In these isolates, the low level of *hmwA *transcript corresponded to the absence of any HMW-reactive protein by Western blotting (only one band in strain 91 and no reactive-bands in strain 72). Although the presence of antigenically variant HMW adhesins, which lacked the epitopes recognized by our MAbs, could not be ruled out, the absence of reactivity even with the 28D polyclonal antiserum (which exhibits a broad cross-reactivity among different NTHi isolates) suggests that one or both HMW proteins were actually down-modulated in strains 91 and 72, respectively.

In the study performed by Dawid and colleagues, no strain 12 variants possessing a number of repeats < 15 were found and analyzed [11]. In our study, the invasive NTHi strains 56 and 91 were found to contain 9 and 13 repeats, respectively, within the *hmw2A *and *hmw1A *promoters. In these strains, the level of *hmwA *transcript was either similar (strain 56/*hmw2A*, 9 repeats) or higher (strain 91/*hmw1A*, 13 repeats) than that of the strain12/*hmw1A *reference mRNA, indicating that decreasing repeat number below 15 does not negatively affect the level of gene expression.

The inverse relationship between increasing number of repeats and the amount of mRNA expression [11] was not always confirmed in our NTHi isolates: this trend was observed for the *hmw2A *but not for the *hmw1A *gene. In fact, strain 56 showing, within the *hmw1A *promoter, two repeats more than strain 91 and one repeat less than the reference strain 12, exhibited the highest level of expression of the *hmw1A *gene, suggesting the presence of additional mechanisms of control contributing to modulation of the transcript level of this gene. Further studies are needed to address this point.

The role in pathogenicity of NTHi infection of such a fine-tuned modulation of the expression of the HMW adhesins is still not clear. As previously observed by other authors, expression does not switch on or off but involves multiple stages ranging from very weak to very strong, with a series of gradations in between [11]. It has been speculated that phase variation mechanism of HMW1 and HMW2 adhesins plays a fundamental role in enabling the organism to survive in different environments [11]. Although we analyzed only a few invasive NTHi isolates, results of the present study suggest that expression of both HMW proteins is not essential for bacterial survival in sterile sites, since two of the three isolates tested had at least one down-regulated HMW adhesin. Actually, inspection of the *hmw1A *and *hmw2A *promoter regions of other 10 invasive NTHi isolates from our collection seemed to confirm this hypothesis since 5 out of 10 possessed a high number of repeats (> 17 repeats) in both *hmwA *promoters, 2 isolates contained a high number of repeats in at least one *hmwA *promoter and only 3 had a number of repeats close to 15 (corresponding to the maximum level of transcript) in both *hmwA *promoters (data not shown). Monitoring of *hmwA *gene expression in vivo could provide insight into the actual role of the HMW adhesins in the different phases of invasive NTHi infection, being this issue of great interest in view of the potential use of these adhesins as vaccine components. On the other hand, it is well known that both hypervariable expression and heterogeneity of surface antigens are tools used by microorganisms to evade host defenses. According to previous data, *hmwA *genes are characterized by a high level of polymorphism in the receptor binding domains that are surface exposed and subject to selective pressure [9,12,13]. Variable expression of HMW adhesins provides a further mechanism to escape host immune system.

## Conclusion

To conclude, our study demonstrates for the first time a variation in expression of the *hmwA *genes in clinical NTHI isolates other than the prototype strain 12 and confirms that increasing number of 7-bp repeats within *hmwA *promoters generally correlates with decreasing levels of mRNA transcript. However, the results herein reported also suggest that additional mechanisms of control may contribute to regulation of HMW protein expression, especially in modulating the transcript level of the *hmw1A *gene. Further studies aimed to identify environmental conditions able to influence the *hmwA *genes transcript profiles both in vitro and during natural NTHi infection are needed.

## Methods

### Bacterial strains and growth conditions

Three invasive NTHi isolates (strains 56, 72 and 91 isolated from pleural fluid, blood and peritoneal fluid, respectively) previously found to possess both *hmw*1A and *hmw*2A genes [9]were analyzed. The original strains had been passaged no more than three times before being collected for DNA and RNA extractions. NTHi strain 12 (kindly provided by S. J. Barenkamp, St. Louis University, MO), from which the *hmw1A *and *hmw2A *genes were originally cloned [6], was also included in the study as reference strain. Bacteria were grown overnight on chocolate agar plates supplemented with Vitox (Oxoid Ltd., Basingstoke, Hampshire, UK) at 37°C in 5% CO_2_. For RNA extractions, bacterial strains were grown in Brain Heart Infusion (BHI) broth enriched with Haemophilus Test Medium Supplement (Oxoid Ltd.) and incubated under the same conditions.

### Analysis of repeat number within the *hmw1A *and *hmw2A *promoters

In each isolate, individual colonies grown on chocolate agar plates were picked and boiled for preparation of total DNA. Amplicons including the *hmw1A *and the *hmw2A *promoter regions were obtained by two consecutive PCRs. The full length *hmw1A *and/or *hmw2A *genes were separately amplified using primers and following PCR conditions previously described [9]. The resulting PCR products were then used as templates in a nested PCR reaction employing primers flanking the promoter region (REP3 5'-GCAGTCTATATGCAAATATT-3' and REP4 5'-TTCTTGCCCCTCCCTCCCTT-3'), designed on the strain 12 DNA sequences [GenBank: U08875, GenBank:U08876]. The nested amplification was performed using 1.5 U of Takara *Taq *(Takara Bio Inc., Shiga, Japan) with a reaction mixture containing buffer (10 mM Tris-HCl, 50 mM KCl, and 1.5 mM MgCl_2_), 2.5 mM of each deoxynucleoside triphosphate, 50 pmol of each primer and 2 μl of template in a total volume of 50 μl. Thermocycling conditions were: 95°C for 5 min, 25 cycles of 95°C for 30 s, 50°C for 30 s, and 72°C for 30 s, followed by a final elongation step of 72°C for 5 min. Amplification gave rise to expected PCR products of approximately 250 bp. PCR fragments were purified using the QIAquick PCR purification kit (QIAGEN S.p.A, Hilden, Germany). Sequencing was performed on both strands by using the fluorescent dideoxy-chain terminator method on an ABI 3730 DNA sequencer (Applied Biosystems, Foster City, CA, USA).

### RNA extraction

Total cellular RNA was extracted from mid-log phase grown NTHi strains 12, 56, 72 and 91 by using the RNeasy mini Kit (QIAGEN S.p.A.). To eliminate genomic DNA traces, total RNA was incubated with 20 U of RNase-free DNase (QIAGEN S.p.A.) for 20 min at 25°C on the RNeasy columns, according to the manufacturer's instructions. The quantity and the quality of extracted total RNA was estimated by UV spectrophotometry and by electrophoresis on 1.0% native agarose gels. To verify possible DNA contamination, extracted RNA was subjected to q-RT-PCR as described below but in absence of reverse transcriptase. No amplification product was obtained.

### qRT-PCR

To distinguish between *hmw1A *and *hmw2A *genes, primers for qRT-PCR were designed within the core-binding domain regions exhibiting a good level of discrimination between the two genes [6,12]. Since the *hmw1A *and *hmw2A *core-binding domains showed some degree of heterogeneity at DNA sequence level among the strains analyzed [9], a specific primer set for each isolate was designed by using DNAMAN sequence analysis software (version 5.2; Lynnon Corp., Quebec, Canada) with the exception of strains 56 and 12 sharing both identical *hmw1A *and *hmw2A *core-binding domain sequences and primer set (Table 2).

**Table 2:** qRT-PCR primers used in this study

Primer set	Strain/gene amplified	Nucleotide sequence (5' to 3')	Reference sequence	Size, bp
gyrAfrw	All/*gyraseA*	GCGTGTTGTGGGTGATGTAA	L42023	82
gyrArev		GTTGTGCCATACGAACGATG		
hmw1Afrw	Hi12, Hi56/	CCGGTGGTTTTGTGGAGACGTCG	M84616	133
hmw1Arev	*hmw1A*	TGAAGTATTGCTGCGTCCTG		
hmw2Afrw	Hi12, Hi56/	CCGGTGGTTTTGTGGAGACATCG	M84615	121
hmw2Arev	*hmw2A*	GCGAAGGGGGTCTTCGGCTTCA		
72.1Afrw	Hi72/	CCGGTGGTTTCGTGGAGACATCA	AJ937359	184
72.1Arev	*hmw1A*	GCGTTCAGGTTCATTTGATGCACTTTCTA		
72.2Afrw	Hi72/	CCGGTGGTTTCGTGGAGACATCA	AJ937360	178
72.2Arev	*hmw2A*	GACATTATTGTTATAGATTGTTTCTGTGGTGTA		
91.1Afrw	Hi91/	CTGGCGGCTTTGTGGAAACGTCA	AJ920372	174
91.1Arev	*hmw1A*	GAGCTCTCGGTACCTAGACCAGT		
91.2Afrw	Hi91/	ACTGGTGGTTTTGTGGAGACATCAGG	AJ937352	158
91.2Arev	*hmw2A*	CTTATTGGGGTTATCGCCTCTAGATATT		

qRT-PCR was performed by using each primer set with the SuperScript III Platinum SYBR green One-Step qRT-PCR kit (Invitrogen Life Technologies Corp.) in a LightCycler 2.0 system (Roche, Mannheim, Germany). The housekeeping *gyraseA *(*gyrA*) gene was used as internal standard gene for RNA quantity normalization. The qRT-PCR mixture (total volume 20 μl) contained 1.0 μl One-step enzyme mix, 10.0 μl Syber green 2× (MgSO_4 _3 mM), 1.0 μl BSA 20× and 0.25 μM of each primer. The PCR conditions were as follows: a reverse transcriptase step at 50°C for 2 min, a denaturation step at 95°C for 2 min, followed by 35 amplification cycles, each of 95°C for 5 s, 55°C for 10 s and 72°C for 10 s. Quantitative standard curves were obtained by amplification of an internal fragment of the *gyrA *gene using as templates ten fold serial dilutions of known amounts (100 ng/μl, 10 ng/μl, 1 ng/μl, 0.1 ng/μl, and 0.01 ng/μl) of genomic DNA from the reference strain12. Standard curve was produced using the LightCycler 2.0 software (Roche). Three independent experiments, each performed in triplicate, were carried out for each *hmwA *gene from each NTHi isolate.

### Detection of HMW proteins by Western blotting

Whole-cell proteins were prepared and resolved by sodium dodecyl sulfate (SDS)-polyacrylamide gel electrophoresis (PAGE) on a 7.5% acrylamide gel, as previously described [9]. After proteins were transferred to nitrocellulose sheets, HMW proteins were detected by using either the 28D rabbit polyclonal antiserum or the 3D6 and/or AD6 mouse immunoglobulin G MAbs. All polyclonal and monoclonal antibodies were kindly provided by S. J. Barenkamp, St. Louis University, MO, USA. The 28D polyclonal antiserum raised against the HMW1 and HMW2 proteins of NTHi strain 5, another previously analyzed strain expressing both [6], is broadly cross-reactive with the HMW proteins of other NTHi strains (S. J. Barenkamp, personal communication). 3D6 and AD6 MAbs recognize both the HMW1 and HMW2 proteins, although the AD6 MAb appears to react better with HMW2 [14,15]. The epitope recognized by 3D6 MAb is not known, while that recognized by AD6 MAb is localized within the 75 aminoacid segments at the carboxy termini of the HMW1 and HMW2 proteins [14]. The 28D rabbit polyclonal antiserum was used in a 1:250 dilution; the 3D6 and AD6 MAbs were 1:25 diluted. Anti-rabbit immunoglobulin G alkaline phosphatase conjugate was used as the secondary antibody (Sigma Aldrich Corp., St. Louis, MO. USA). NTHi strain 12 was used as a control of HMW1 and HMW2-expressing isolates.

## Abbreviations

NTHi: nontypeable *Haemophilus influenzae*; qRT-PCR: quantitative real-time reverse transcription-PCR.

## Competing interests

The authors declare that they have no competing interests.

## Authors' contributions

MG: Carried out molecular biology experiments; participated in writing the manuscript. AC: Guidance and supervision throughout the qRT-PCR experiments; participated in writing the manuscript. RC: Carried out the immunoassays. PM: critical reading and amendment of parts of the manuscript. MC: conceived the study design, coordinated the teamwork and wrote the manuscript. All authors read and approved the final manuscript.
